# Robot-assisted closed reduction of femoral shaft fractures: a prospective controlled study

**DOI:** 10.1007/s00264-025-06623-z

**Published:** 2025-08-01

**Authors:** Chunpeng Zhao, Honghu Xiao, Qiyong Cao, Mingjian Bei, Bo Li, Yingchun Song, Gang Zhu, Xinbao Wu

**Affiliations:** 1 Beijing Jishuitan Hospital, Beijing, China; 2 Rossum Robot Co., Ltd., Beijing, China

**Keywords:** Robot, Femoral shaft fracture, Closed reduction, Prospective

## Abstract

**Purpose:**

To evaluate and compare the effectiveness of an intelligent fracture reduction robotic system in assisting closed reduction and intramedullary nailing of femoral shaft fractures with that of conventional fluoroscopy-assisted manual reduction and fixation.

**Methods:**

In this prospective, non-randomized controlled study, 30 patients with newly diagnosed femoral shaft fractures were enrolled, with 15 cases in the experimental group (robot-assisted) and 15 cases in the control group (conventional). The experimental group utilized an orthopaedic surgical navigation system to assist in closed reduction and intramedullary nailing, while the control group underwent fluoroscopy-assisted manual reduction and fixation. The reduction time, total operation time, intraoperative fluoroscopy count, blood loss, and reduction error were compared between the two groups.

**Results:**

Baseline characteristics were similar across both groups. The experimental group required significantly fewer intraoperative fluoroscopies (36.67 ± 25.41 vs. 117.26 ± 61.28, *P* < 0.001). Postoperative femoral length discrepancy (1.74 ± 1.37 mm) and anteversion difference (3.66 ± 3.37°) were significantly smaller in the experimental group compared to the control group (4.16 ± 2.67 mm, *P* = 0.004; 13.81 ± 9.58°, *P* = 0.001). Intraoperative blood loss was comparable between groups (experimental group: 207.33 ± 119.91 mL vs. control group: 240.00 ± 139.13 mL, *P* = 0.497). Reduction time was not statistically significant (experimental group: 74.27 ± 27.38 min vs. control group: 69.73 ± 34.10 min, *P* = 0.691).

**Conclusions:**

The robot-assisted approach provided more precise fracture reduction, required fewer intraoperative X-ray fluoroscopies, and offered significant advantages over the conventional method for the minimally invasive treatment of femoral fractures.

## Introduction

Femoral shaft fractures, which occur between the lesser trochanter and the femoral condyle, are among the most common fractures in adults, accounting for approximately 6% of all fractures [1, 2]. These fractures typically result from traffic accidents, sports injuries, or falls in elderly individuals [2]. Femoral shaft fractures are associated with a higher incidence of complications, such as poor healing, delayed union, and nonunion, compared to fractures in other locations. Malunion or nonunion of the fracture can lead to severe disability, reduced quality of life, and increased treatment costs [3, 4]. In the United States, the direct costs of medical care and productivity loss within the first six months post-injury are estimated to be as high as $23,000 per limb fracture [5].

Intramedullary nailing is the gold standard for treating adult femoral shaft fractures, as it reduces the risk of implant failure, refracture, and wound complications such as infection. Additionally, it provides stable fixation with micro-movements at the fracture site, which promotes callus formation [6]. Closed reduction with intramedullary nailing preserves the blood supply to the fracture ends, thus facilitating healing. However, the procedure is technically challenging [7, 8]. Achieving a successful closed reduction and intramedullary nailing requires precise nail entry point selection, accurate reduction of the fracture ends, appropriate implant choice, and secure distal locking. Due to the thick soft tissue and muscular coverage in the thigh, femoral fractures are difficult to access during closed reduction. The surrounding muscle mass, along with significant fracture displacement, creates considerable resistance, making manual reduction particularly challenging [9].

To assist with fracture reduction, various techniques have been developed, including lever reduction, auxiliary instruments, small incisions, intramedullary reduction, strapping, and blocking nails. While these methods can aid in reduction, they still require multiple fluoroscopic images, and in some cases, continuous manual reduction under fluoroscopy. This increases radiation exposure for both the physician and the patient, prolongs surgery duration, and complicates the maintenance of proper reduction.

In collaboration with Beijing Rossum Robotics Technology Co., Ltd. and Beijing Jishuitan Hospital, a novel pelvic fracture closed reduction robot system was developed, incorporating an elastic traction technique to reduce the required reduction force [10, 11]. This innovation successfully reduced the robot’s required force to a manageable level, enabling autonomous reduction during surgery. The system has been recognized as the first of its kind in clinical practice, and its development has been presented at prominent robotics conferences such as IROS [12], and published in high-impact surgical journals [13]. To date, the system has been used in over 200 surgeries and clinical trials involving 92 cases.

Building on our prior work, we explored the system’s feasibility in a preliminary cadaver study of eight femoral specimens [14], where robotic assistance achieved consistent reduction with minimal deviations in length and anteversion while reducing radiation exposure and preserving peri-fracture blood supply. To confirm these promising results in a clinical setting, we subsequently initiated the present prospective, non-randomized controlled trial.

## Materials and methods

### Study design

This prospective, non-randomized controlled study included 30 patients with fresh femoral fractures, divided into two groups: 15 patients in the experimental group and 15 patients in the control group. In the experimental group, patients underwent fracture reduction and internal fixation using the intelligent fracture reduction robot system. In the control group, patients received traditional fluoroscopy-guided manual reduction and fixation.

### Inclusion and exclusion criteria

Inclusion Criteria were: (1) Age between 18 and 80 years, regardless of gender; (2) Closed femoral shaft fractures requiring reduction and fixation; (3) Patients who understood the benefits and risks of the study and provided written informed consent.

Exclusion Criteria were: (1) Severe open femoral fractures.; (2) Bilateral fractures with significant displacement, preventing the use of orthopaedic surgical navigation; (3) Unstable vital signs or patients unable to tolerate anaesthesia or surgery; (4) Poor skin conditions or infections at the planned implant insertion site; (5) Systemic diseases such as severe bleeding disorders, heart disease, or respiratory conditions; (6) Pathological fractures (e.g., primary or metastatic tumours) or revision surgeries (e.g., malunion, nonunion, or infection); (7) Patients deemed unsuitable for the study by the investigator for other reasons.

### Intelligent fracture reduction robot system

**Fig. 1:**
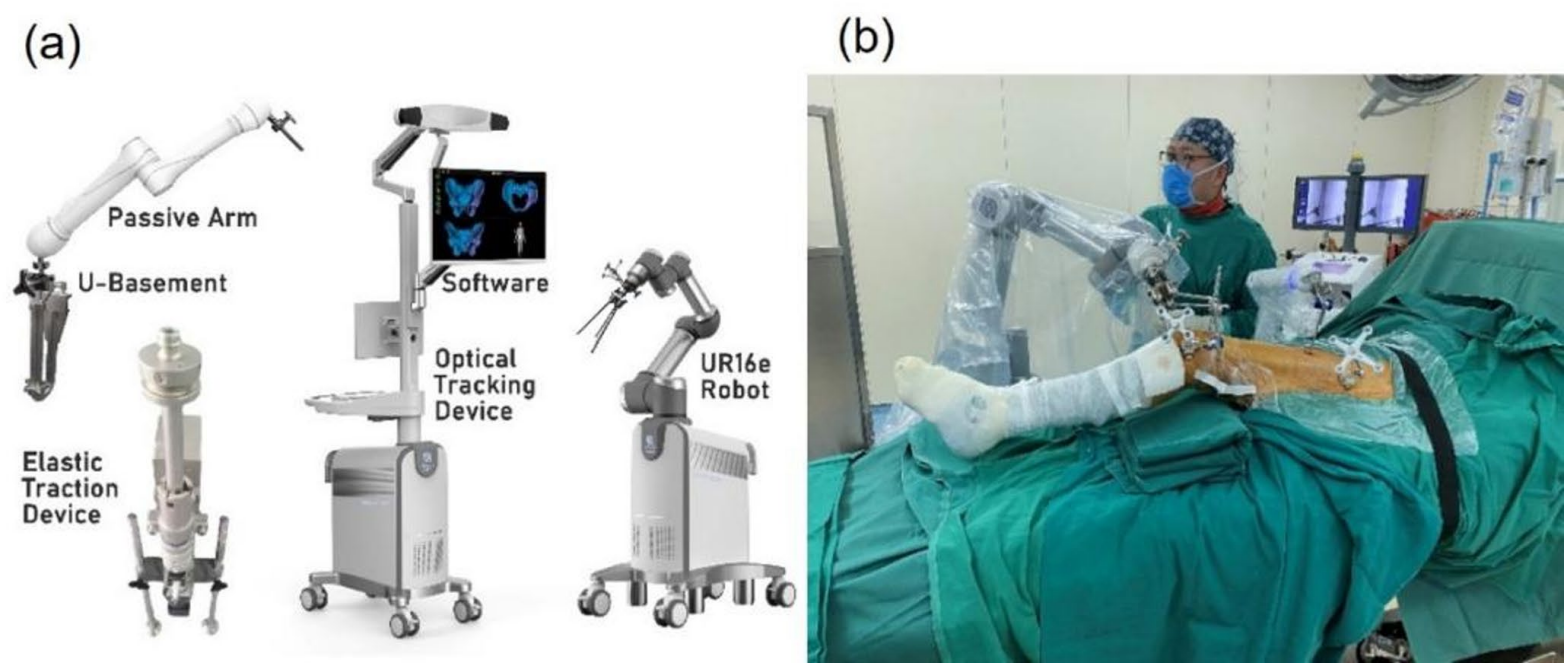
Robot-assisted reduction system. (**a**) Schematic illustration of the robot system components; (**b**) Application of the robot system in the operating room

The intelligent fracture reduction robot system consists of two main components (Fig. 1):

#### 3D vision-based system

This includes femoral fracture reduction software (comprising reduction planning and intraoperative registration/navigation software), a robotic arm (UR16e), and an optoelectronic tracking device (NDI Polaris Vega with optical tracking tools).

#### Auxiliary robot structure

This comprises a passive holding arm installed beside the operating table and an elastic traction device located at the table’s end. The proximal femur is stabilized using a nine-degree-of-freedom electrically controlled passive arm (Pass Arm 1, Rossum Robot Co., Ltd., RPC), while the elastic traction device provides quantified traction force to counteract the constraint force of surrounding soft tissues.

### Surgical procedure in the experimental group

All procedures were performed by at least 15 years of experience traumatic specialist (C.P. Zhao and X.B. Wu), employed conventional fluoroscopy-guided closed reduction techniques for the control group.

#### Preoperative planning

**Fig. 2:**
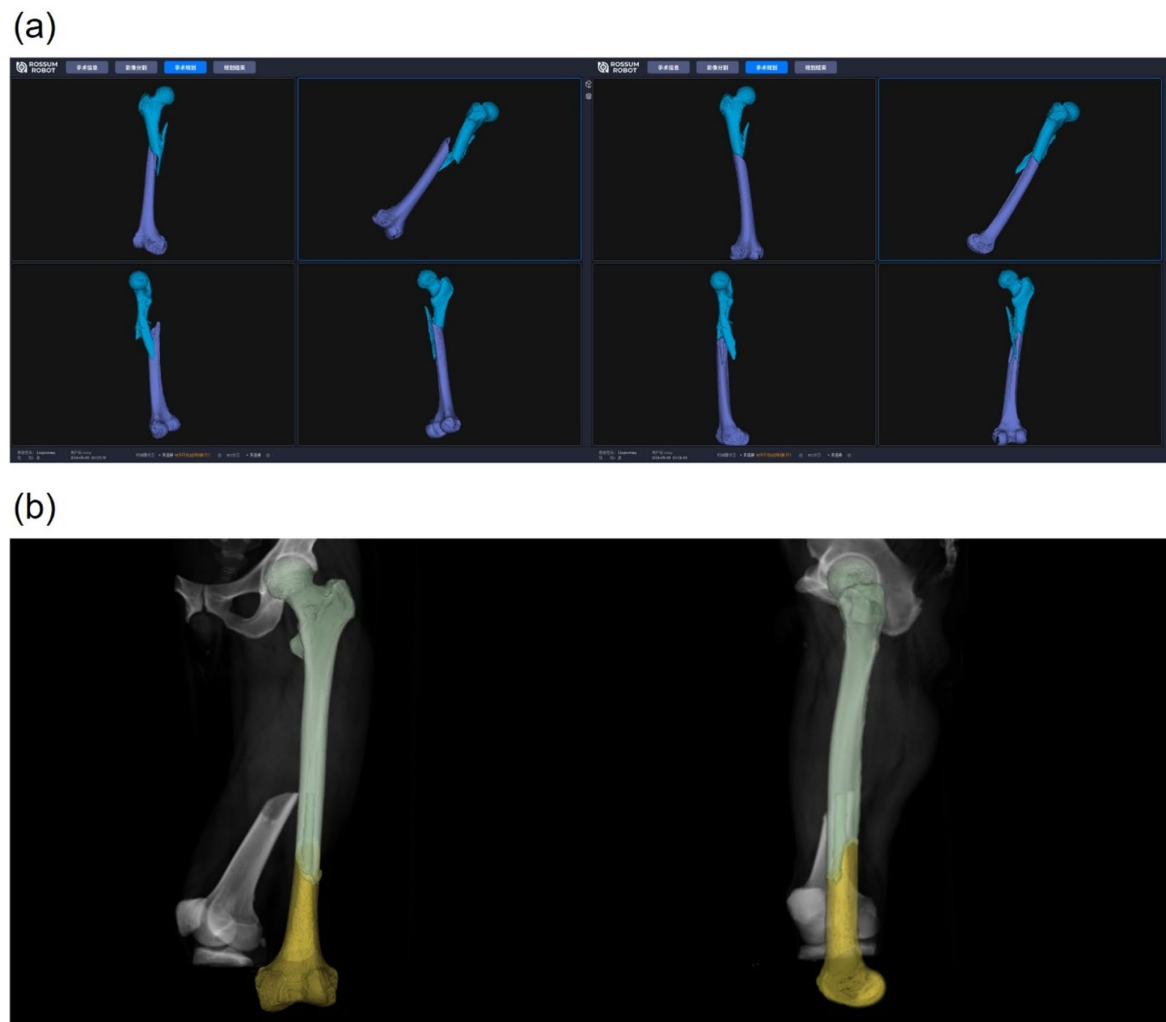
Automated reduction planning and simulation. (**a**) Segmentation and 3D reconstruction of the proximal and distal femoral fragments; (**b**) Planning of the reduction position and simulation of the reduction trajectory

CT images of the femoral shaft fracture were segmented and reconstructed using specialized software, which planned the final reduction position (Fig. 2). The surgeon confirmed the fracture reduction pathway to avoid any collisions or interlocking during the procedure.

#### Intraoperative registration and navigation

**Fig. 3:**
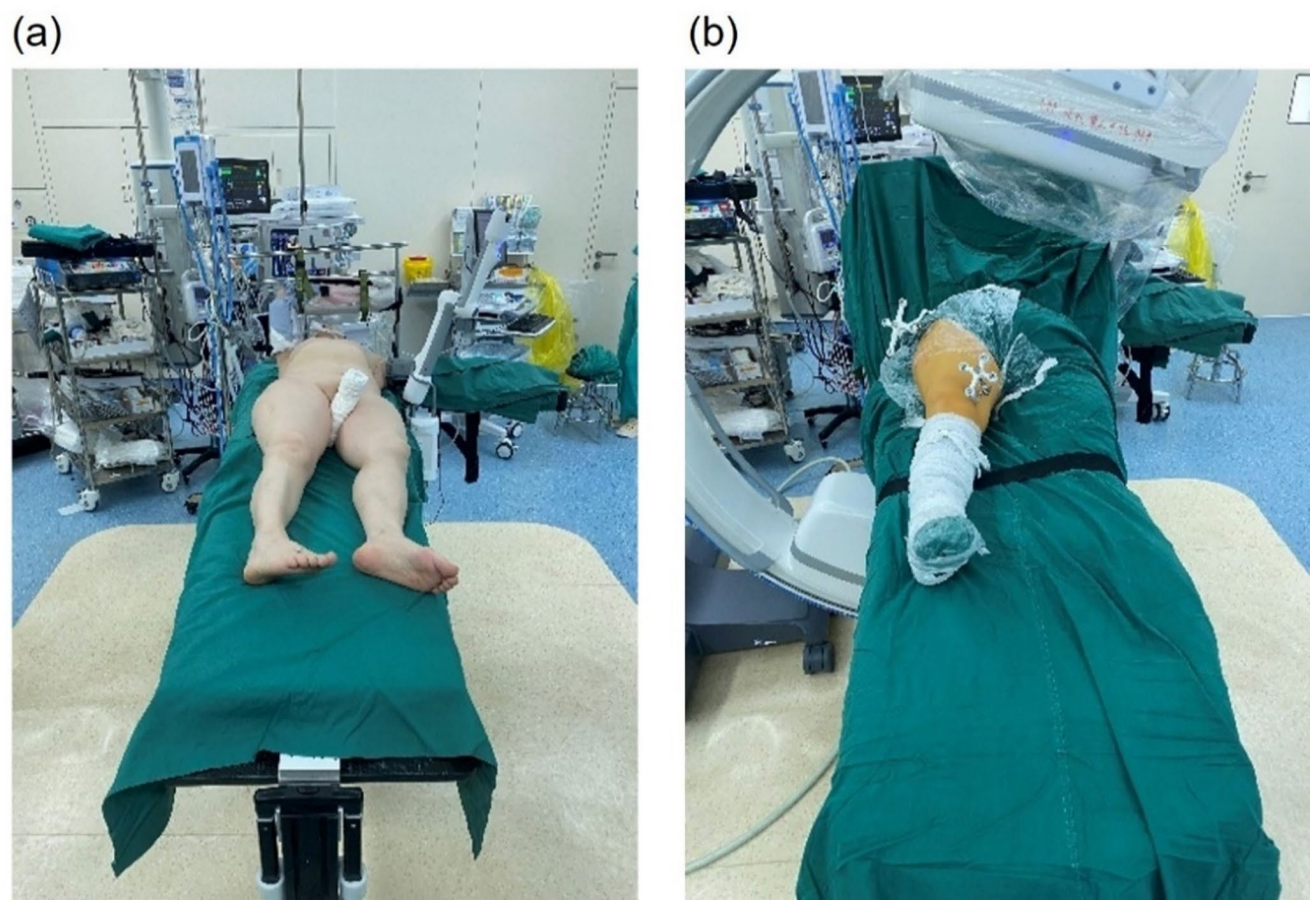
Intraoperative setup of the robotic-assisted reduction system. (**a**) Patient positioning for robotic-assisted fracture reduction; (**b**) Placement of skeletal trackers at the proximal and distal fracture sites

**Fig. 4:**
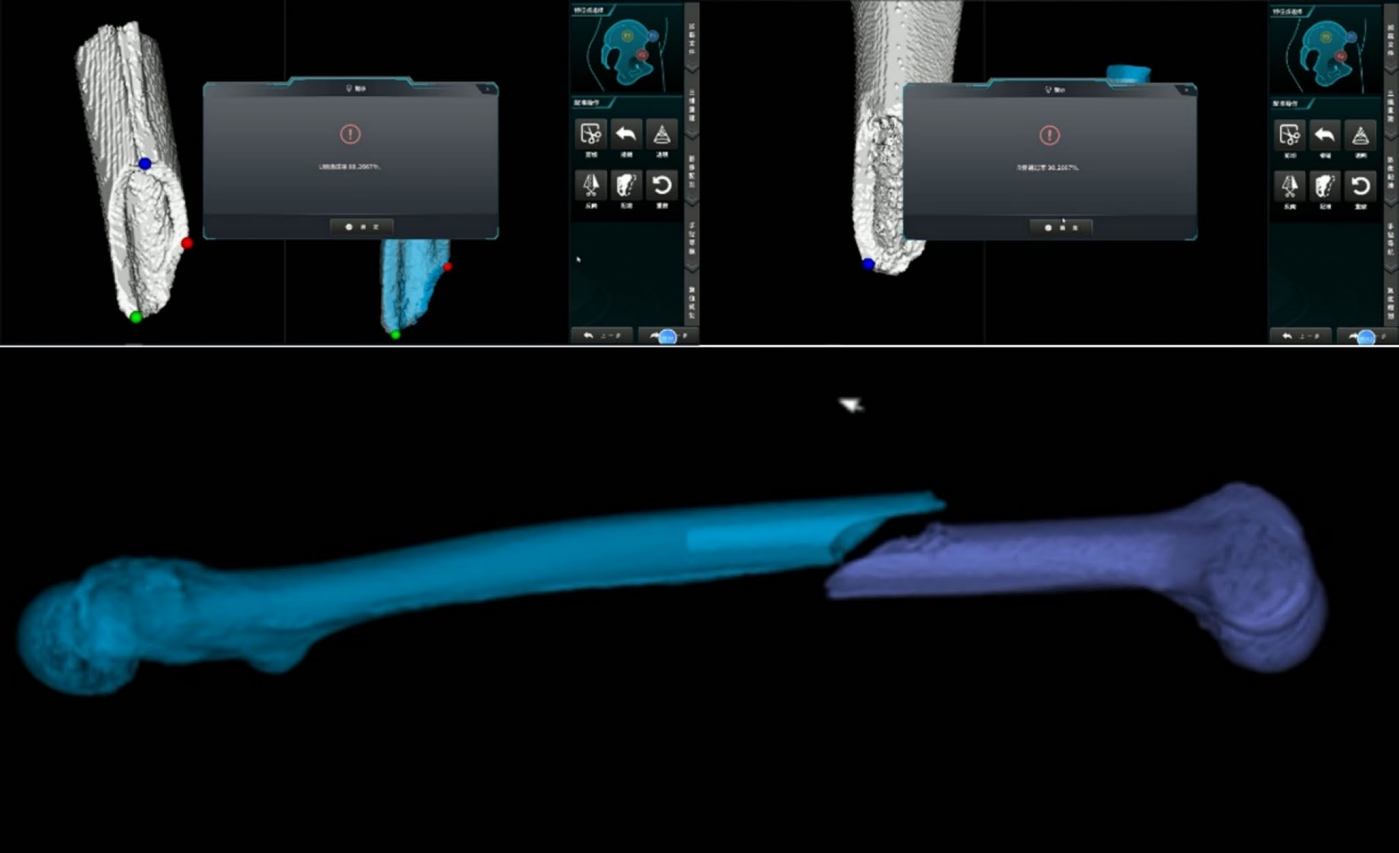
Registration of intraoperative CBCT and preoperative CT images for precise fracture alignment

The patient was positioned supine on a radiolucent carbon fiber surgical table (Fig. 3), with the affected limb adducted and internally rotated. A perineal post was used to counteract traction. Skeletal trackers were placed on the proximal and distal ends of the fracture. The proximal tracker was fixed with a 2.5 mm Kirschner wire (K-wire), and the distal tracker was placed on the anterior surface of the medial femoral condyle. After the trackers were placed, a cone-beam CT (CBCT) scan was performed and registered with preoperative CT images (Fig. 4). The time from tracker fixation to registration completion was recorded as the registration time.

**Fig. 5:**
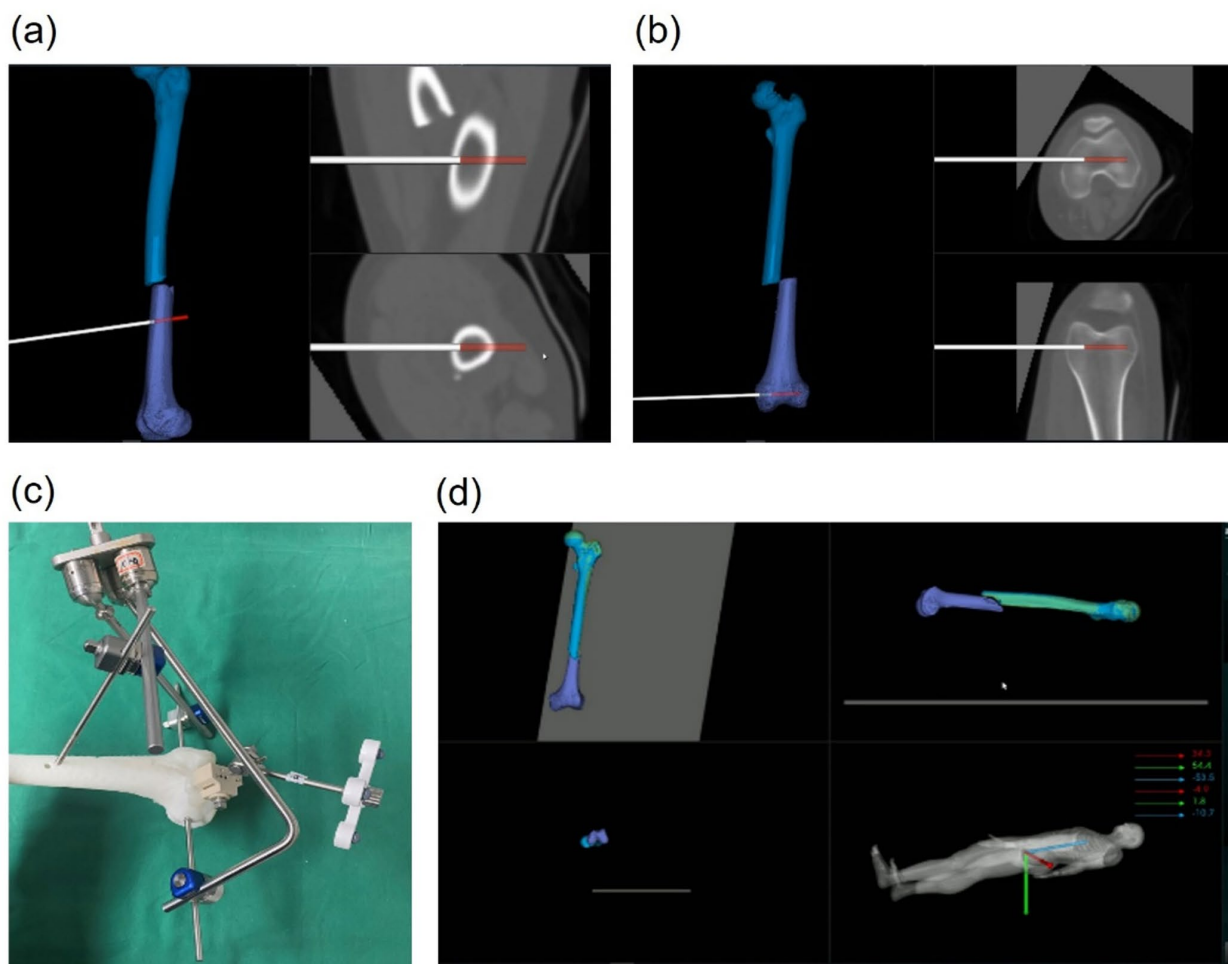
Intraoperative navigation and reduction process. (**a**) & (**b**) Navigation-guided insertion of femoral holding pins; (**c**) Schematic of holding pin placement at the distal fracture site: one pin is perpendicular to the anterior cortex, and the other is perpendicular to the sagittal plane, penetrating the medial and lateral femoral condyles; (**d**) Automated completion of fracture reduction under navigation monitoring

A passive arm was installed on the healthy side of the patient to stabilize the proximal femur (Fig. 1). Following image registration, holding needles were inserted into the proximal and distal fracture ends using the navigation interface (Fig. 5). These needles were connected to the passive arm and robotic gripper, with the traction system applied to provide necessary force during reduction. The robotic arm then performed the fracture reduction under real-time 3D navigation. The time from holding needle insertion to successful reduction was defined as the motion time. The total reduction time was calculated by adding the registration time and motion time (Table 1). After reduction, the guide pin was inserted, the medullary canal reamed, the intramedullary nail inserted, and locking completed.

### Surgical procedure in the control group

In the control group, a surgeon performed conventional fluoroscopy-guided closed reduction, following standard manual techniques.

### Primary outcomes

#### Reduction quality assessment

**Fig. 6:**
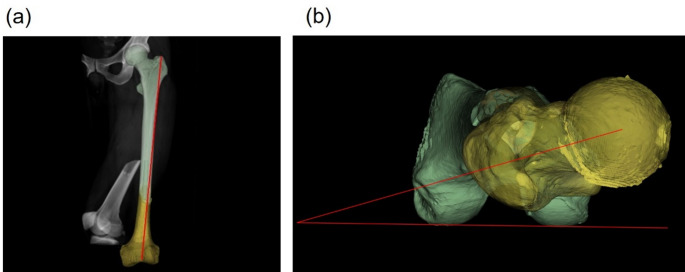
Measurements of femoral length and neck anteversion angle. (**a**) Measurement of femoral length (L0) from the apex of the greater trochanter to the apex of the intercondylar notch after planned reduction; (**b**) Measurement of the femoral neck anteversion angle (FNA0) after planned reduction based on the CT Reikerås method, defining the femoral neck axis and projecting the angle onto the simulated femoral shaft CT axial slice

Postoperative CT imaging was performed. The femoral shaft length and anteversion angle were measured using the Reikerås method [15]. The preoperative and postoperative measurements of femoral length and anteversion angle were compared to calculate the reduction length difference and rotational difference (Fig. 6).

### Secondary outcomes

The duration of surgery, reduction time, number of fluoroscopies, and blood loss were compared between the two groups.

### Statistical analysis

All analyses were conducted in SPSS (IBM Corp., Armonk, NY). Continuous variables were assessed for normality via the Shapiro–Wilk test. Variables with normal distributions are presented as mean ± SD and compared by Student’s t-test; non-normal variables are presented as median (Q1–Q3) and compared by Mann–Whitney U test. Categorical variables are expressed as counts (percentages) and compared using χ² or Fisher’s exact test as appropriate. Two-sided *P* < 0.05 was considered statistically significant.

## Results

### Demographic data

The age range for patients in the experimental group was 18–70 years, while in the control group it was 15–74 years. The average age was similar between the two groups (experimental group: 39.4 ± 16.97 years, control group: 39.67 ± 16.22 years). The sex distribution was also comparable (experimental group: 60% male, control group: 53% male). The average time from injury to surgery was similar between the groups (experimental group: 8.0 ± 4.44 days, control group: 6.6 ± 4.66 days, Table 1).

### Outcome data

The number of intraoperative fluoroscopies in the experimental group (36.67 ± 25.41 times) was significantly fewer than in the control group (117.27 ± 61.28, *P* < 0.001). Intraoperative blood loss was smaller in the experimental group (207.33 ± 119.91 mL) compared to the control group (240.00 ± 139.13 mL, *P* = 0.497). The average fracture reduction time in the experimental group (74.27 ± 27.38 min) was slightly longer than in the control group (69.73 ± 34.10 min, *P* = 0.691). The total surgery time in the experimental group (189.60 ± 68.86 min) was significantly longer than in the control group (142.33 ± 54.08 min, *P* = 0.046) (Table 1).

The intelligent fracture reduction robot system, using optoelectronic tracking and intraoperative navigation, ensured precise reduction with minimal invasiveness. Postoperative CT scans confirmed that the femoral length and rotation were restored to clinically acceptable levels. The postoperative femoral length difference in the experimental group (1.74 ± 1.37 mm) and the femoral anteversion angle difference (3.66 ± 3.37°) were significantly smaller than in the control group (4.16 ± 2.67 mm, *P* = 0.004, and 13.81 ± 9.58°, *P* = 0.001, Table 1).

**Table 1:** Demographics and operative data

	Experimental Group (*N* = 15)	Control Group (*N* = 15)	*P*-Value
**Demographics**			
Age (mean ± SD, years)	39.40 ± 16.97	39.67 ± 16.22	0.965
Gender			
Male	9 (60%)	8 (53%)	-
Female	6 (40%)	7 (47%)	-
Time Interval from Injury to Surgery (mean ± SD, days)	7(5,10)	6(4,8)	0.260
Fracture Classification, n (%)			0.953
A1	2 (13.33%)	1 (6.67%)	-
A2	3 (20%)	3 (20%)	-
A3	6 (40%)	3 (20%)	-
B1	0 (0%)	1 (6.67%)	-
B2	1 (6.67%)	3 (20%)	-
B3	2 (13.33%)	2 (13.33%)	-
C1	0 (0%)	0 (0%)	-
C2	0 (0%)	1 (6.67%)	-
C3	1 (6.67%)	1 (6.67%)	-
**Operative data**			
Length Difference (mean ± SD, mm)	1.70(0.80,1.90)	4.15(2.20,6.00)	0.009
Rotation Difference (mean ± SD, degree)	2.40(1.40,5.70)	14.0(4.5,19.0)	0.001
Blood Loss (mean ± SD, mL)	200(100,300)	200(200,300)	0.532
Operation Time (mean ± SD, mins)	189.60 ± 68.86	142.33 ± 54.08	0.046
Fracture Reduction Time (mean ± SD, mins)	74.27 ± 27.38	69.73 ± 34.10	0.691
X-ray Frequency (mean ± SD, n)	23(18,58)	104(74,124)	< 0.001

## Discussion

Surgical robots, known for their precision, stable operation, reproducibility, fatigue resistance, and immunity to radiation, have been increasingly applied in orthopaedics. These systems assist orthopaedic surgeons in performing various procedures with greater accuracy, particularly in trauma, spine, joint, sports medicine, and orthopaedic oncology surgeries [16].

Fracture reduction robots face significant challenges, as they must withstand substantial loads during procedures and require ample space to execute reduction tasks effectively [17]. As a result, many of these robotic systems remain in the prototype phase. For instance, the University of Regensburg in Germany developed a femoral shaft fracture reduction robot, RepoRobo, based on the Staubli RX130 industrial robot [18]. Similarly, Hannover Medical School, in collaboration with Braunschweig University of Technology, created a reduction robot system utilizing master-slave control with force and position feedback [19]. Additionally, the University of Tokyo and Osaka University in Japan developed FRAC-Robo, a traction robot that aids femoral fracture reduction by applying traction via a boot connected to the patient’s foot [20]. Another example is RAFS, an image-guided robotic system proposed by Aston University for the percutaneous treatment of intra-articular knee fractures [21].

In China, several institutions have made strides in robotic fracture reduction. For example, Tzu Chi Hospital in Taiwan, in collaboration with National Taipei University and Chang Gung University, developed a robotic system that controls knee flexion, applies separate traction to the thigh and calf, and rotates the foot [22]. Beihang University has created a vision-based, teleoperated system that uses preoperative CT scans for high-precision minimally invasive fracture reduction [23]. Additionally, Beijing Jishuitan Hospital, in partnership with Tianzhi Navigation Technology Co., Ltd., developed a six-degree-of-freedom fracture reduction robot based on master-slave control [24].

Our research team has also developed an intelligent fracture reduction robot system, which has completed successful clinical trials involving 92 pelvic fracture cases. Building on this success, we are continuing to enhance this system for the closed reduction of long bones. Crucially, the robotic strategy for pelvic fracture reduction leverages mirroring technology. In this approach, the intact hemipelvis serves as a virtual template, guiding the fractured side towards a predefined mirrored anatomical position. Within our system, this mirroring protocol essentially defines the anatomical endpoint for pelvic reduction [17]. Conversely, the robotic reduction protocol for long bone fractures, exemplified by the femur, employs a statistical shape model (SSM). This model, constructed from a database of normative anatomical variations, calculates the optimal reduction by comparing the current fracture configuration against statistical norms, thereby directing the reduction towards an idealized, statistically derived anatomical alignment. The endpoint for long bone reduction is determined by the convergence of fracture fragments towards the position defined by the SSM. Furthermore, the inherent biomechanical and anatomical challenges encountered in these two regions are markedly different [25]. Pelvic reduction frequently encounters difficulties with locking between fracture fragments [26]. In contrast, the primary challenge in long bone reduction lies in achieving and maintaining a stable reduction and purchase at the proximal and distal fracture fragments. This often necessitates meticulous control over the robotic arm’s force application and spatial manipulation within the typically constrained surgical workspace to effectively control and align the bone segments during the reduction maneuver. These fundamental distinctions are elaborated upon in greater detail within the Discussion section.

In our approach, we import CT data into the robotic software, where it undergoes 3D reconstruction to plan the reduction of the distal ends of the fracture using the mirror principle and the engagement relationships of the fracture surfaces. This ensures that no collisions or obstructions occur during the reduction process. Additionally, we have observed significant morphological differences in the bilateral femurs of the same patient, such as variations in the anteversion angle and femoral length. These findings align with those of Dimitris Dimitriou et al. [27].

For the fracture reduction procedure, we employed a method where the fixation pin is inserted until it penetrates the cortex, ensuring it does not interfere with subsequent proximal reaming or the insertion of locking screws. This approach maintains stability for the tracker without affecting the planned surgical steps. The distal tracker is placed on the lateral femoral condyle, near the edge of the patella, to avoid entering the patellofemoral joint. This positioning minimizes the likelihood of interference during the insertion of the intramedullary nail. Following tracker placement, we conduct a cone-beam CT (CBCT) scan and integrate the scanned data into the robotic system for registration.

To ensure the alignment and orientation of the fracture reduction, we align the distal end with the proximal end, accounting for potential sagittal plane adjustments. The software calculates the position of the proximal end in advance, providing the surgeon with guidance to prevent collisions during the procedure. To hold the proximal end in place, a passive arm is attached near the patient’s head, locking the proximal femur in position. Once registration is complete, the holding pins are implanted at both ends of the fracture. For the proximal end, a holding pin is inserted from front to back, featuring a large threaded design to enhance stability. This pin is monitored in real-time to ensure it does not encroach upon the medullary cavity, ensuring that subsequent reaming and screw insertion proceed smoothly.

For the distal holding, two pins are connected to the robotic system via a three-pronged connector. One pin passes perpendicularly through the femoral condyles in the sagittal plane, positioned just above the femoral condyles to avoid interfering with the locking screws. The second pin is inserted similarly to the proximal holding pin. All pins are carefully monitored during insertion to avoid damage to intra-articular tissues, with the procedure being guided by the navigation drill.

Once both distal pins are connected, the robotic system performs the fracture reduction automatically, and fluoroscopy is used to verify the alignment. Unlike traditional methods, where an assistant must manually apply traction, our elastic traction device replaces this need. This device offers continuous, real-time adjustment of traction force during the reduction, providing more stable and efficient assistance throughout the procedure.

In summary, our intelligent fracture reduction robot system offers a highly precise, minimally invasive approach to femoral shaft fractures. The robotic arm, operating under real-time monitoring, effectively maintains the reduction position, providing advantages over traditional methods by reducing the need for intraoperative fluoroscopies.

### Limitations

While this study demonstrates the feasibility of robot-assisted closed reduction for femoral shaft fractures, the small sample size limits the generalizability of the results. Larger trials are needed to confirm the effectiveness of this robotic system in clinical settings. Furthermore, while the system provides navigation and positioning for fracture reduction, its role in subsequent procedures, such as intramedullary nail placement, requires further investigation. Future studies should assess the accuracy and operability of these additional functions to validate their clinical utility.

### Interpretation

This study explores the use of an intelligent surgical robotic system for femoral shaft fractures (Chinese Patent No.: 202110779036.4). The results show that the system can intelligently plan the reduction position, design the reduction path, and provide real-time 3D navigation during surgery. The robotic arm successfully completes the reduction as per the planned path, with the resulting reduction quality and accuracy surpassing conventional manual reduction techniques. Notably, the number of fluoroscopies required was significantly lower compared to traditional methods. As research progresses, we believe this intelligent fracture reduction system will offer a novel approach to the minimally invasive treatment of femoral shaft fractures and potentially other long bone fractures.

## Conclusion

The intelligent, minimally invasive fracture reduction robot is capable of autonomously planning the reduction path for femoral fractures. Using a robotic arm under real-time monitoring, it performs and maintains a minimally invasive reduction. Compared to traditional methods, this approach achieves more precise fracture reductions, requires fewer intraoperative fluoroscopies, and offers significant advantages for the minimally invasive treatment of femoral fractures.

## Data Availability

No datasets were generated or analysed during the current study.
